# A Review Concerning the Polysaccharides Found in Edible and Medicinal Plants in Xinjiang

**DOI:** 10.3390/molecules28052054

**Published:** 2023-02-22

**Authors:** Hailiqian Taoerdahong, Gulimila Kadeer, Junmin Chang, Jinsen Kang, Xiaoli Ma, Fei Yang

**Affiliations:** College of Pharmacy, Xinjiang Medical University, Urumqi 830011, China

**Keywords:** plants, polysaccharide, pharmacology

## Abstract

Approximately 110 types of medicinal materials are listed in the Chinese Pharmacopoeia, both for medicinal purposes and for use as food. There are several domestic scholars who have carried out research on edible plant medicine in China and the results are satisfactory. Though these related articles have appeared in domestic magazines and journals, many of them are yet to be translated into English. Most of the research stays in the extraction and quantitative testing stage, and there are a few medicinal and edible plants that are still under in-depth study. A majority of these edible and herbal plants are also highly enriched in polysaccharides, and this has an effect on immune systems for the prevention of cancer, inflammation, and infection. Comparing the polysaccharide composition of medicinal and edible plants, the monosaccharide and polysaccharide species were identified. It is found that different polysaccharides of different sizes have different pharmacological properties, with some polysaccharides containing special monosaccharides. The pharmacological properties of polysaccharides can be summarized as immunomodulatory, antitumor, anti-inflammatory, antihypertensive and anti-hyperlipemic, antioxidant, and antimicrobial properties. There have been no poisonous effects found in studies of plant polysaccharides, probably because the substances have a long history of use and are safe. In this paper, the application potential of polysaccharides in medicinal and edible plants in Xinjiang was reviewed, and the research progress in the extraction, separation, identification, and pharmacology of these plant polysaccharides was reviewed. At present, the research progress of plant polysaccharides in medicines and food in Xinjiang has not been reported. This paper will provide a data summary for the development and utilization of medical and food plant resources in Xinjiang.

## 1. Introduction

There is a long history of the discovery, practical application, and cultivation of medicinal plants in China, which is one of the countries with the greatest medicinal plant resources. There are several areas within the country, in areas populated by ethnic minority groups, where medicinal plant resources are abundant. This lays the foundation for the development of ethnic medicine, such as Uygur medicine, Mongolian medicine, Tibetan medicine, and so on.

**Figure 1:**
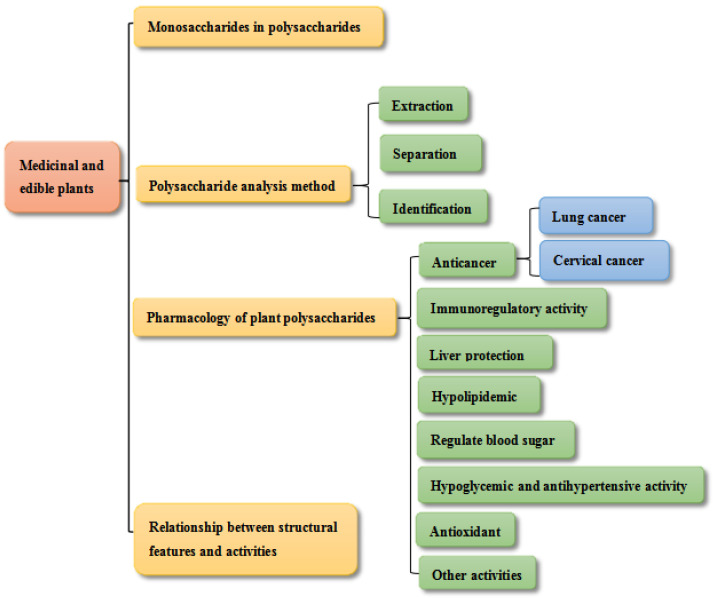
Content distribution map.

**Figure 2:**
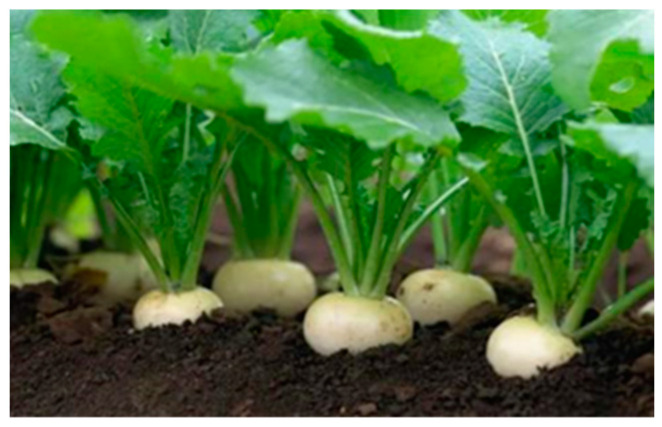
*Turnip* (*Brassica* L.).

**Figure 3:**
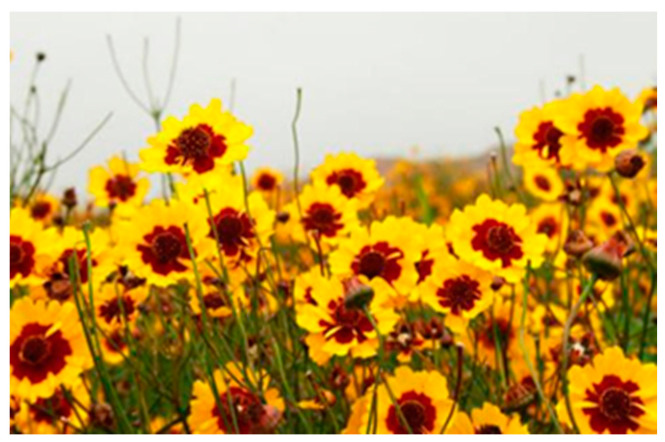
*Kunlun snow chrysanthemum* (*Coreopsis tinctoria* Nutt.).

**Figure 4:**
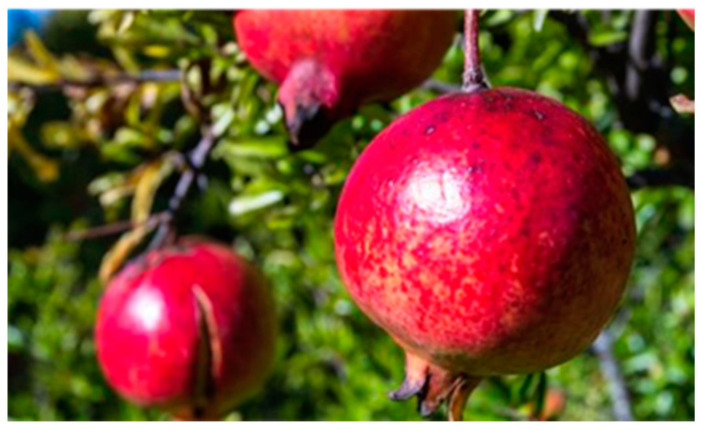
*Pomegranate* (*Punica granatum* L.).

**Figure 5:**
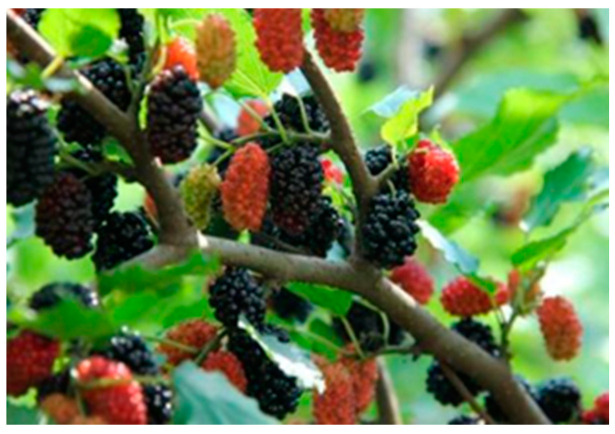
*Mulberry* (*Morusalbal*.).

**Figure 6:**
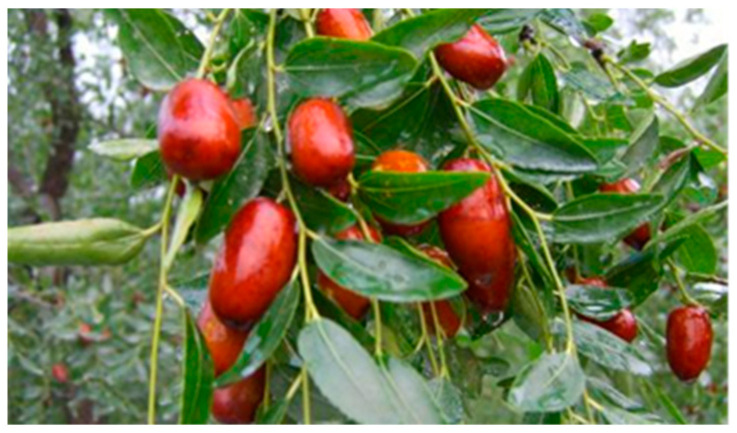
*Jujube* (*Ziziphus jujuba* Mill.).

The concept of “homology of medicine and food” is actually a reflection of the thoughts of dietary therapy, medicinal diet, and health preservation in traditional Chinese medicine, reflecting the traditional Chinese understanding of the connection between the origins of medicine and food. The theory of “homology of medicine and food” has existed in traditional Chinese medicine since ancient times. This theory maintains that many foods are both food and medicine. In ancient, primal societies, the taste and medicinal effects of various foodstuffs and medicines were gradually discovered as people hunted for food and realized that many foodstuffs could serve both as medicines and as foods. It was tricky to strictly distinguish between the two. This is the basis of the theory of “homology of medicine and food”, and food therapy. Plant polysaccharides are a high polymer constructed through the dehydration of over ten monosaccharide molecules, so that glycosidic bonds form. They have a variety of biological functions: anti-oxidation, anti-tumor, and anti-virus properties, as well as regulating immunity, lowering blood lipid, and so on [1]. The glycosidic bonds of polysaccharides can be divided into α type and β type, and the common glycosidic bonds are α-1,4; β-1,3; β-1,6; and α-1,6. Most of the polysaccharides with outstanding biological activity have β (1→3)-D-glucan main-chain structure. In the early investigation of active substances in traditional Chinese medicine, polysaccharides were usually thought as inactive substances. Once the immunomodulatory effect of fungal polysaccharides was discovered, however, people began to explore the structure and function of polysaccharides [2,3]. As research continues at an increasing pace, it has also been pointed out that phyto-polysaccharides come from a wide range of sources and are versatile in biological activities. Plant-derived polysaccharides are a new forage additive, with characteristic properties of being nonpoisonous, benign, nonresidual, and potent in function. Nowadays, the active biological functions and mechanisms of plant polysaccharides have been studied with increasing attention. The effects and mechanisms of polysaccharides from plants are discussed in this research. Figure 1 shows the content distribution map of this review. In what follows, we summarize the five types of polysaccharides in medicinal and edible plants in Xinjiang, and we show their pharmacological properties, Figure 2, Figure 3, Figure 4, Figure 5 and Figure 6.

## 2. Monosaccharides in Polysaccharides

There are significant differences in the biological activities of polysaccharides composed of different kinds of monosaccharides and glycosidic bonds. Therefore, the composition of monosaccharides and the type and location of glycosidic bonds are not only the basis and premise of the study of polysaccharides, but also an important part of further exploring their structure–activity relationship. Monosaccharide composition has a significant effect on the biological activity of polysaccharides. As shown in Table 1 and Table 2, most of the Xinjiang medicinal and edible plant polysaccharides are composed mainly of arabinose (Ara), galactose (Gal), glucose (Glc), rhamnose (Rha), mannose (Man), xylose (Xyl), and fucose (Fuc). Among these, dextran (D-Glc homopolymer) has been proven to have a strong improvement effect on inflammatory diseases [4], and those polysaccharides with high fucose content often show significant anti-inflammatory activity [5]. It can be seen from Table 1 and Table 2 that the polysaccharides with (1-3), (1-4), and (1-6) glycosidic bonds generally show certain biological activities, and the contents of Gal and Glc are relatively high. Therefore, it can be speculated that the anti-inflammatory effects of these two plant polysaccharides may be related.

## 3. Polysaccharide Analysis Method

**Figure 7:**
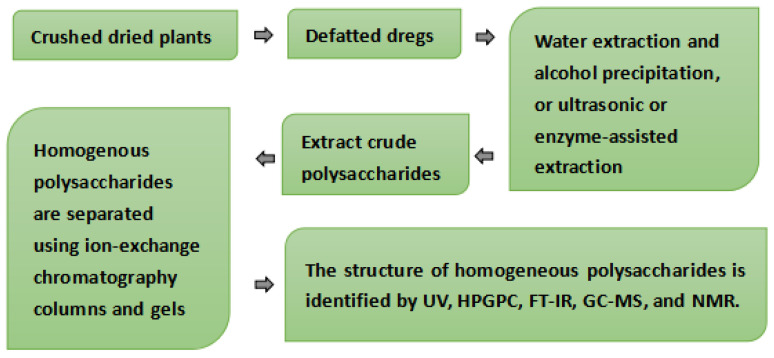
Extraction, isolation, and characterization of polysaccharides found in edible and medicinal plants in Xinjiang.

Polysaccharides are bonded to cell walls or interstitial materials by hydrogen or ionic bonds. Generally, different extraction methods are used depending on the specific components of particular polysaccharides [43,44,45,46,47,48,49,50]. At present, the method of water extraction and alcohol precipitation is generally used in the extraction of polysaccharides. The crude extracted plant polysaccharides are separated and purified after removing impurities. The analysis of monosaccharide composition generally starts with acid hydrolysis, so that the glycosidic bonds of polysaccharides are completely broken (Figure 7). This paper summarizes the extraction, separation, and purification methods of five representative medicinal and food plants in Xinjiang, as shown in the Table 3. At present, most of the plant polysaccharides in Xinjiang are extracted by water extraction and alcohol precipitation, and some are extracted by ultrasound and enzyme-assisted extraction.

The main method of separation and purification is the chromatographic column, using macroporous adsorption resin, an ion-exchange chromatography column, and a gel chromatography column to obtain homogeneous polysaccharides. The main methods to determine the structural framework and functional groups of polysaccharides are chemical methods, including methylation analysis, Smith degradation, periodate oxidation, and partial acid hydrolysis. Mass spectrometry is the main method for determining molecular weight, while the structure of polysaccharides is mainly determined using physical methods, including nuclear magnetic resonance spectroscopy, infrared spectroscopy, ultraviolet spectroscopy, organic mass spectrometry, and so on, as shown in Figure 7. However, it has been observed that few studies have reached the stage of structural identification, and among those that have, many have only studied the primary structure of polysaccharides. A large number of pharmacological studies use crude extracts or partially purified polysaccharides for their research.

## 4. Pharmacological Activity

As researchers study the effects of polysaccharides in pharmacology, the complex and multifaceted activities of polysaccharides in biology have gradually been acknowledged by human beings. Polysaccharides in medicinal and edible plants have become a hot topic in recent years. There are various studies of their effects on immune regulation, tumor cell inhibition, hormone regulation, metabolism, inflammation, and fatigue [56]. As shown in Table 4, active polysaccharides have a certain range of molecular weights, and polysaccharides with excessive molecular weight generally exhibit no activity. If the molecular weight of polysaccharides is too large, this is not conducive to biological activity because it cannot cross the cell membrane into the organism, but if the molecular weight is too low, there is also no activity [19,20]. The acidic polysaccharides in turnips, which have lower molecular weights, have stronger anti-tumor properties, while the neutral polysaccharides of turnips, which have higher molecular weights, exhibit relatively low activity [wujing]. However, this finding is only based on speculation in existing studies; other biological activities and pharmacological effects of the polysaccharides in medicinal and edible plants need to be further studied.

### 4.1. Anticancer

#### 4.1.1. Lung Cancer

It has been shown that turnip polysaccharides have inhibitory effects on Lewis lung cancer in mice [57]. Although the mechanism by which turnip polysaccharides inhibit lung cancer is not clear, it has obvious therapeutic effects on lung cancer.

#### 4.1.2. Cervical Cancer

Medicinal mulberry polysaccharide (PBMF) has a significant inhibitory effect on the proliferation of HeLa cells and is dose-dependent. PBMF induced HeLa cell cycle stagnation in G0/G1 phase. The relative expression of caspase-3 was upregulated to induce early apoptosis and late apoptosis. PBMF can inhibit the proliferation of and induce apoptosis of human cervical cancer HeLa cells, and its mechanism is related to the activation of caspase-3 [58]. The general decrease in caspase-3 expression in cervical cancer cells indicates that the imbalance of cell apoptosis and proliferation induces the occurrence of tumors. However, plant polysaccharides can inhibit the expression of caspase-3 by up-regulating Bcl-2. Based on the above research results, the key substances of plant polysaccharides that inhibit tumor growth, and the mechanism of up-regulation and down-regulation of caspase-3, need to be further studied.

#### 4.1.3. Other Cancers

Studies have shown that total polysaccharides from Kunlun snow chrysanthemums have obvious inhibitory effects on colon cancer [59]. Jujube polysaccharides had obvious inhibitory effects on S180 sarcoma and Eich’s ascites tumors in mice [60]. The mulberry polysaccharide MFP 90-2 has been applied in the preparation of anti-ovarian cancer and anti-pancreatic cancer drugs. MFP 90-2 showed better inhibitory activity on OVCAR-3 cell proliferation and migration, in addition to an obvious dose-effect relationship and time dependence [61]. As natural extracts, plant polysaccharides of medicinal and edible origin are abundant in raw materials and easy to obtain, and their preparation methods are mature and stable, which lays a foundation for the development and application of safe, low toxicity, and efficient antitumor drugs.

### 4.2. Immunoregulatory Activity

In the RAW264.7 cell model, it was found that when the concentration of Xinjiang turnip pectin polysaccharides ranged from 12.5 μg/mL to 100 μg/mL, the proliferation activity and phagocytosis capacity of the cells were significantly increased, the release of NO, TNF-α, IL-1β, and IFN-γ cytokines was effectively stimulated, and the dose-effect was obvious. The results showed that pectin polysaccharides from Xinjiang turnips had strong immunomodulatory activity [12]. Some researchers have analyzed the immunomodulatory effect of pomegranate peel polysaccharides (PPP) on immunosuppressed mice induced by cyclophosphamide (CTX), and they have found that PPP could be used as an efficacious adjacent immunopotentiating therapy or an alternative means of lessening chemotherapy-induced immunosuppression, and that it could also be utilized as an immunostimulant for food and pharmaceutical industries [61]. It was found that the transformation and resistance of lymphocytic cells in mice were enhanced, and the level of hemolysin in the blood was increased. It has an immune protection effect [52]. Mulberry polysaccharides (MLP) can restore the body weight of CTX-treated mice, improve immune response, balance the expression of inflammatory cytokines, and prevent hepatotoxicity. In addition, MLP intervention can promote the production of SCFAs and regulate intestinal flora. These results indicate that polysaccharides from mulberry leaves have the potential to be a functional food for intestinal immune regulation, and they provide a theoretical basis for the application of polysaccharides from mulberry leaves in fortified food and the further development and utilization of mulberry leaves [62].

### 4.3. Liver Protection

Studies have shown that kunlun snow chrysanthemum polysaccharides (KSCP) can up-regulate the expression of the anti-apoptosis-related protein Bcl-2, down-regulate the expression of the pro-apoptotic proteins caspase-3 and Bax, and increase the ratio of Bcl-2/Bax in liver cells, suggesting that KSCP may exert its hepatoprotective effect by inhibiting apoptosis of liver cells. KSCP can reduce the levels of ALT and AST in the serum of mice with CCL4-induced liver injuries, and histopathological observation of the liver also confirmed that KSCP can reduce the severity of hepatocyte enlargement, inflammatory infiltration, and necrosis in mouse liver tissue. At the same time, KSCP can reduce the level of MDA and increase the level of SOD in the liver tissue of mice, suggesting that it can improve the oxidative stress level of the body [63]. Shu Guangwen et al. found that polysaccharides from mulberry could down-regulate the levels of TNF-α, IL-LP, and IL-6 as well as the expression of NF-κB p65 protein in liver tissue, and that they can inhibit the occurrence of inflammatory liver reactions. They have a protective effect on acute liver injury [63]. Other studies have investigated the mulberry’s total polysaccharide (MFP) and its protective effects against APAP-induced acute liver injury in mice, and the possible mechanism of protection. MFP was found to protect against APAP-induced acute liver injury in mice, which was related to enhancing the antioxidant capacity of the liver and inhibiting the inflammatory response of the liver. Expression of anti-inflammatory and inflammatory cytokines such as IL-1, TNF-α, IL-6, and IL-8 triggers inflammation, and is one of the reasons for impaired hepatocyte function as well as islet-cell-damage-induced diabetes [64,65]. It has been found that plant polysaccharides also mostly exert various kinds of influence by regulating cytokines.

### 4.4. Hypolipidemic Activity

SD rats were fed with pomegranate fruit polysaccharides in concentrations of 75, 150, and 300 mg/kg, once a day for 28 days. After treatment, the levels of blood glucose, TC, TG, HDL-C, and LDL-C in each group were compared. The results showed that pomegranate fruit polysaccharides could effectively reduce blood glucose and regulate blood lipids in diabetic rats. Mulberry leaf polysaccharide (MLP) has obvious inhibitory effects on pancreatic lipase (PL). MLP could significantly inhibit the development of lipid accumulation in HepG2 cells. MLP also significantly decreased the expression of digestive enzymes in the pancreas. According to the above studies, the anti-obesity effect of MLP may be caused by inhibiting lipid absorption by reducing the activity of PL [66,67].

### 4.5. Hypoglycemic and Antihypertensive Activity

Mulberry leaf polysaccharides upregulate the expression of antiapoptotic Bcl-2 protein and mRNA in diabetic rats, inhibit the activation of the proapoptotic protein caspase-3, stimulate insulin secretion from islet cells, increase insulin content, and reduce blood glucose [68]. Polysaccharide from mulberry decreased the normal blood pressure of Sprague–Dawley rats by increasing the production of endothelial nitric oxide (NO), and induced dependent relaxation of the enteric mesangial arteries and the inner skin of rats, thus regulating blood pressure [69]. The y-aminobutyric acid, gluside, and mistletoe in mulberry leaves are known to reduce blood pressure. The y-aminobutyric acid in mulberry leaves is a neurotransmission material, which can promote the metabolism of brain tissue and restore brain cell function, and at the same time can improve brain blood flow, enhance the activity of the angiotensin-converting enzyme I, and promote the decline of blood [70]. When the dosage of purified polysaccharide from turnip was 400 mg/kg of body weight, the blood glucose of tested mice decreased after 3 days. After 6 and 9 days, blood glucose decreased significantly. Turnip polysaccharide showed significant hypoglycemic effect [45].

### 4.6. Antioxidant

The Kunlun snow chrysanthemum and 21 other different cultivated types of medicinal chrysanthemum were investigated for their ability to scavenge OH and DPPH free radicals. The experimental results show that polysaccharide from Kunlun snow chrysanthemum exhibits effective removal of OH and DPPH free radicals, and that it more effectively removes DPPH free radicals than other types of medicinal chrysanthemum. By studying the scavenging ability of Kunlun snow chrysanthemum polysaccharides on OH and DPPH free radicals, it was found that the scavenging ability of Kunlun snow chrysanthemum polysaccharides increased with the increase in polysaccharide concentration, second only to vitamin C [71]. Mulberry polysaccharides can inhibit the formation of brown lipids in the intestines of mice, improve swallowing frequency and transport energy, prolong the life of mice, and delay the aging of mice [25]. Lipotoxicity is considered a potential cause of metabolic syndrome, and currently, there is no effective treatment. The protective effect of the black mulberry polysaccharide, BP1, on lipid toxicity of HepG2 cells induced by palmitic acid (PA) was stronger than that of BP2 and BP3. BP1 can eliminate ROS accumulation, improve mitochondrial function, reverse glutathione consumption, improve antioxidant enzyme activity, and effectively reduce PA-induced lipid toxicity. BP1 activated the Nrf2 signaling pathway, a master regulator of the antioxidant defense system, through increasing Nrf2 nuclear translocation and phosphorylation. Collectively, these results demonstrate that BP1 has great potential for applications in lipid disorders [72]. Several studies have suggested that polysaccharides, p-coumaric acid, and p-coumaric acid-β-D-glucopyranoside in turnips are potentially active components contributing to the anti-hypoxic effects of turnips [50]. Plant polysaccharides can reflect antioxidant activity through the upregulation of antioxidant enzymes (GSH, SOD, and CAT), as well as by downregulating malondialdehyde (MDA), restoring total antioxidant capacity, reducing kidney damage caused by oxidative stress, and improving blood glucose levels. Therefore, the various activities of plant polysaccharides are correlated.

### 4.7. Other Activities

In the study of structural characteristics and anti-fatigue effects of Brassica L. acidic polysaccharide (BRAP), the anti-fatigue effect of turnip polysaccharide was evaluated by using a mouse weight-bearing swimming model. The levels of serum (brain) urea nitrogen (BUN), lactic acid (LA), and malondialdehyde (MDA) in mice were significantly decreased by turnip polysaccharides [73]. The serum levels of inflammatory mediators were significantly reduced in Wistar rats after gavage of the turnip polysaccharides, at 5 and 2.5 mg/kg in each group [74]. The serum level of inflammation mediators decreased in both the low-dose and high-dose conditions, compared with those in model controls [75]. Turnip polysaccharide can effectively inhibit the secretion of various inflammatory factors in asthmatic rats and reduce the occurrence of inflammatory reactions. Other plants have exhibited similar activity with respect to liver-glycogen-content determination and serum levels, also measured by mouse loaded-swimming tests. The results showed that polysaccharides from mulberry could prolong the loading swimming time of mice, decrease the level of urea nitrogen in serum, and increase the glycogen reserve in the liver after swimming, which collectively had a certain anti-fatigue effect [72]. The effects of polysaccharides from Mulberry leaf (MLP-1) on STZ-induced diabetic nephropathy (DN) were studied. After 8 weeks of instillation, the levels of serum insulin, fasting blood glucose, urinary albumin, urinary protein, and connective tissue growth factor were significantly reduced in STZ-induced DN rats. These results indicate that MLP-1 can effectively inhibit renal fibrosis and has the potential to treat DN [75].

## 5. Relationship between Structural Features and Activities

The biological activity of plant polysaccharides is mainly to make use of their similar structure to produce immune activity through the mechanism of immune regulation, as shown in Table 4. The primary structure and advanced structure of polysaccharides are closely related to their biological activity, which is also a hot spot in the research field of polysaccharides. Like oligosaccharides, polysaccharides have first-order, second-order, third-order, and fourth-order structures, but at present, the structural studies of these five kinds of plant polysaccharides are mainly in the analysis of their primary structure.

The primary structure of plant polysaccharides is closely related to their biological activity. Most of the polysaccharides with outstanding biological activity are connected by (1-3) glycosidic bonds, with a main chain structure of β-(1-3)-D-glucan [76,77], some side chains, a certain degree of branching, as well as some special functional groups connected to the glycosyl group. Experimental evidence shows that most sulfated polysaccharides have obvious antiviral, anti-tumor, and anticoagulant activities, including glucan sulfate, pentosan sulfate, and so on. For example, sulfation modification can greatly improve the antioxidant and hypoglycemic activity of jujube polysaccharides, while polysaccharide selenate from pomegranate seeds can significantly slow down the increasing trend of POV value of vegetable and animal oils, showing a certain anti-oxidation ability [78]. However, merely having the same or similar main chain structures is not enough to make polysaccharides have high biological activity [79,80]. Differences in polysaccharide molecular structure can affect their activity; for example, wolfberry polysaccharide results show that LBP-a8, LBP-a3, LBP-a1, and LBP-a4 have the activity to inhibit SMMC-7721 cell proliferation, and that this effect is concentration- and time-dependent, but LBP-a8 promotes the growth of human SMMC-7721 cells, while LBP-a4 is found have anticancer activity.

**Table 4:** Summary of five polysaccharide contents and related diseases.

Plants	Molecular Weight of Polysaccharides	Polysaccharides	Disease and Function	Experimental Subject	Mechanism	Reference
*Turnip*	7590~6080 Da and 4751~6873 Da	8.99%	Inflammation	RAW 264.7 macrophage	Regulate NF-κB, TNF-α proteins	[81]
			Regulate blood sugar level	SD rat	Promote hepatic glycogen synthesis and suppress its decomposition	[82]
			Asthma	Wistar rat	Control TNF-α, IL-6, IL-2, and CRP level	[75]
			Lung cancer	Lewis lung cancer mice	-	[56]
*Kunlun snow chrysanthemum*	8200~8700 Da and 6100~6500 Da.	8.96%	Acute liver injury	KM mouse	Affects tumor necrosis factor-α (TNF-α) and interleukin-6 (IL-6) level	[83,84]
			Anti-colon cancer	HCT116 cells, CACO-2 cells	Apoptosis	[58,85]
*Pomegranate*	14~1400 kDa	Skin: 27.3% Leaf: 1.809% Seed: 2.65%	Lipid lowering	-	Inhibition of cholesterol esterase and pancreatic lipase	[9,18]
			Immunoregulation	RAW 264.7 macrophage	Inhibition of NO release	[86]
			Regulate blood sugar and blood lipids	SD rat	Lower blood glucose and serum TC, TG, and LDL-C, and increase serum HDL-C	[87]
*Mulberry*	80~250 kDa	9.95%	Myocardial inflammatory response	H9C2 cells	Apoptosis	[88,89,90,91,92,93]
			Cancer	MCF-7 cells, Kupffer cells, HeLa cell, MKN-45 cells, NCI-H1650 cells	Affects the content of MDA, NO, IL-6, IL-1 β, and NF-κB, and the viability values of TNF-α	[24,60]
			Alcoholic liver injury	SPF mouse	Intervention of linoleic acid, α-linoleic acid, and glycerophospholipid metabolism	[68]
*Jujube*	45,900, 69,860, and 195,100 Da	3.68%	Diabetes	Mouse	Lower the FBG, TC, and TG in diabetic mice, and increase the HDL content	[53]
			Lipid lowering	Rat	Reduce TG, CHO, LDL-C, and increase the levels of HDL-C. Improve T-AOC, SOD level, and reduce the MDA content	[54,55]
			Cancer	MKN-45	Apoptosis	[55,56]

## 6. Conclusions

In addition to the above-mentioned plants used for both medicine and food, there are many plants in Xinjiang that have significant pharmacological properties and have been developed into products, such as Xinjiang pomegranate emulsions for the prevention and treatment of gynecological diseases, germicidal garlic oil, Cistanche deserticola oral liquid for purification and moistening intestines, etc. The diversity of geographical and climatic conditions in China provides conditions for the growth of various characteristic plants. With the development of modern ethnopharmacology, traditional Chinese medicine, pharmaceutical botany, and other fields, future research will involve ethnic herbs and local characteristic medicine that people often come into contact with in their daily life. This paper summarizes the research results on polysaccharides from several medicinal and edible plants in Xinjiang, and provides a reference for researchers and facilitates the future development of this field. In this paper, the extraction, separation, structure identification, and pharmacological activities of several polysaccharides were studied, and it was found that they have a lot of overlap. It can be seen that they have the activities of regulating immunity, fighting cancer, lowering lipids, regulating blood sugar, and protecting the liver. It is found that the mechanism of action of plant polysaccharides generally exerts various kinds of activities by affecting cytokines, enzymes, and signaling pathways, which are interleaved with each other. Through the composition of polysaccharides, and the proportion of monosaccharides and polysaccharide species, it is found that different sizes of polysaccharides have different pharmacological properties, and that some polysaccharides with special properties will contain special monosaccharide components, such as the Korla fragrant pear polysaccharide and pomegranate skin polysaccharide both containing trehalose, which gives them antibacterial, immune-modulatory, and antioxidant properties. Generally speaking, among these polysaccharides, which are derived from plants used for both medicine and food, the polysaccharides with good biological properties generally have the following structural characteristics: the primary structure has the main chain structure β-(1-3)-D-glucan with moderate branching degree; it has connections to some functional groups in the sugar group; and it falls within a certain range of molecular weight. The above several kinds of polysaccharides from medicinal and edible plants in Xinjiang are mainly extracted by water extraction and alcohol precipitation, but there are also other methods including ultrasonic-assisted extraction, microwave-assisted extraction, and enzyme extraction. The components of polysaccharides are typically analyzed by chromatography (high performance liquid chromatography, gas chromatography, gas chromatography–mass spectrometry, etc.). It has been found that plant polysaccharides have many benefits, so in order to make full use of these polysaccharides’ properties, we should develop the extracted polysaccharide components into drugs to assist clinical treatment, amplify the pharmacological effects by increasing the enrichment of polysaccharides, and make appropriate dosage forms to facilitate their use by consumers.

## Figures and Tables

**Table 1:** Monosaccharide components in polysaccharides from some medicinal and edible plants grown in Xinjiang.

Name	Latin Name	Polysaccharide Components and Structures	Reference
*Kunlun snow chrysanthemum*	*Coreopsis tinctoria* Nutt.	**KSCP1** is composed of Glc, Ara, Gal, and Xyl with a molar ratio of 10.53∶5.02∶4.96∶1 and a molecular weight range of 8200~8700 Da. **KSCP2** is mainly composed of Glc, Ara, and Gal, with a molar ratio of 1∶2.78∶5.07 and a molecular weight range of 6100~6500 Da. Pectin polysaccharides: **CTAP-1:** [→4)GalpA(1→]_n_ [→2)Rhap(1→]_m_ m:n = 1:7 (n and m are numbers of monosaccharides, the same is said below) **CTAP-2:** [→4)GalpA(1→]_n_ [→2)Rhap(1→]_m_ m:n = 1:27	[6]
*Jujube*	*Ziziphus jujuba* Mill.	Crude polysaccharide-1a (HJP-1a) is mainly composed of Ara and Gal with a ratio of 56.93 to 19.99. The average molecular weights of **HJP-2, HJP-3,** and **HJP-4** are 4.590 × 10^4^, 6.986 × 10^4^, and 1.951 × 10^5^ Da, respectively	[1,7,8,9]
*Safflower*	*Carthamus tinctorius* L.	At present, there are 11 kinds of polysaccharides isolated from safflower: **SPS1, SPS2, SPS3, CTP, CTLP-1, CTLP-2, HH1-1, SPSa, SPAW, SF1, and SF2**. **SPS2** is composed of Rha, Ara, Xyl, Man, Glc, and Gal with a molar ratio of 4.44:1.46:4.51:5.82:8.23:19.38. The relative molecular weight of SPS3 is 5.86 × 10^3^, and it is composed of Rha, Ara, Glc, and Gal, with a molar ratio of 2.93:11.19:33.68:3.48. **CTP:** Glc, Gal in a ratio of 6.08:1; **CTLP-1:** Ara, Glc, Gal in a ratio of 6.7:4.2:1; **CTLP-2:** Ara, Glc, Gal in a ratio of 16.76:4.28:1; **HH1-1:** Gal, Ara in a ratio of 54, 9:45.1; SPSa and SPAW: Glc; **SF-1:** Rha, Ara, Xyl, Man, Glc, Gal in a ratio of 2.9:7.5:3.8:1:11.6:8.9; **SF-2:** Rha, Ara, Xyl, Man, Glc, Gal in a rato of 2.9:10.3:4.2:1:5.1:8.5.	[10,11,12]
*Chinese wolfberry*	*Lycium dasystemum* Pojark.	Lycium barbarum polysaccharides (LBP) are composed of Man, Rha, GalA, Glc, Gal, and Ara, with a content ratio of 4.98∶2.93∶8.38∶22.44∶25.38∶35.89. There are 20 kinds of Chinese wolfberry polysaccharides currently found: **LbGp2:** 68,200 Da, Ara:Gal = 4:5. Backbone composed of (1→6)-β-Gal. Branches composed of (1→3)-β-Ara and (1→3)-β-Gal terminated with (1→3)/(1→5)-α-Ara. **LbGp3:** 92,500 Da, Ara:Gal = 1:1. Backbone composed of (1→4)-β-Gal. Branches composed of (1→3)-β-Ara and (1→3)-α-Gal terminated with (1→3)/(1→5)-α-Ara. **LbGp4:** 214,800 Da, Ara:Gal:Rha:Glc = 1.5:2.5:0.43:0.23. Backbone composed of (1→4)-β-Gal. Branches composed of (1→3)-β-Gal terminated with (1→3)-α-Ara and (1→3)-β-Rha. **LBPA3:** 66,000 Da, Ara:Gal = 1.2:1. Heteropolysaccharide with (1→4), (1→6). **LBPB1:** 18,000 Da, Ara:Glc = 1:3.1. Heteropolysaccharide with (1→4), (1→6) β-glycosidic bond. LBP-a4: 10,200 kDa, Fuc:gal = 0.41:1. LBPC2: 12,000 kDa, Xyl:Rha:Man = 8.8:2.3:1. Heteropolysaccharide with (1→4), (1→6) β-glycosidic bond. LBPC4: 10,000 kDa, Glc. Heteropolysaccharide with (1→4), (1→6) α-glycosidic bond. **LBP1a-1:** 115,000 Da, Glc, α-(1→6)-D-glucan. **LBP1a-2:** 94,000 Da, Glc, α-(1→6)-D-glucan. **LBP3a-1**: 103,000 Da, Polygalacturonan with (1→4)-α-glycosidic bond. **LBP3a-1:** 103,000 Da, Polygalacturonan with (1→4)-α-glycosidic bond. **LBP3a-2:** 82,000 Da, GalA, composed of a small amount of Gal and Ara Polygalacturonan with (1→4)-α-glycosidic bond. **LBLP5-A:** 113,300 Da, (1→3)-linked Gal, (1→4)-linked Gal, (1→3)-linked Ara, (1→5)-linked Ara, and (1→2, 4)-linked Rha. **WSP:** Rha:Fuc:Ara:Xyl:Man:Gal:Glc = 1.6:0.2:51.4:4.8:1.2:25.9:7.3. Backbone composed of (1→2)-linked-Rha and (1→4)-linked-Gal. Branches composed of (1→5)-linked-Ara terminated with Ara residues, and (1→4)-linked-Xyl terminated with Man residues. **AGP:** Rha:Ara:Xyl:Gal:Glc:GalA:GlcA = 3.3:42.9:0.3:44.3:2.4:7. Backbone composed of linear homogalacturonan fragments and rhamnogalacturonan fragments. Side chains mainly composed of β-1,6- and β-1,4-galactopyranan and α-1,5-arabinofuranan. **LBP-IV:** 41,800 Da, Rha:Ara:Xyl:Glc:Gal = 1.61:3.82:3.44: 7.54:1. Backbone composed of both α- and β-anomeric configurations of Ara and Glc. Rha was located at terminal of polysaccharide chain. **LbGp1:** 49,100 Da, Ara:Gal = 5.6:1. Backbone composed of (1→6)-Gal. Side chains mainly composed of (1→3)-Gal/(1→4)-Gal and (1→3)-Ara/(1→4)-Ara. Ara was located at terminal of branch. **P-LBP:** 64,000 Da, Fuc:Rha:Ara:Gal:Glc:Xyl:GalA:GlcA = 1.00:6.44:54.84:22.98:4.05: 2.95:136.98:3.35. Backbone composed of (1→4)-α-GalA. Side chains mainly composed of α-1,2- and α−1,4-Rha and α-1,5-Ara. **LBP1B-S-2:** 80,000 Da, Rha:Ara:Gal:Glu = 3.13:53.55:39.37:3.95. Backbone composed of 1, 3-linked β-D-Gal and 1, 6-linked β-D-Gal. Branches contain 1, 4-linked β-D-GlcA; T-linked β-D-Gal; 1, 6-linked β-D-Gal; T-linked α-L-Ara; T-linked β-L-Ara; 1, 5-linked α-L-Ara; and T-linked β-L-Rha. **LRGP1:** 56,200 Da, Rha:Ara:Xyl:Man:Glu:Gal = 0.65:10.71:0.33:0.67:1:10.41. Backbone composed of (1→3)-linked Gal. The branches were composed of (1→5)-linked Ara, (1→2)-linked Ara, (1→6)-linked Gal, (1→3)-linked Gal, (1→4)-linked Gal, and (1→2,4)-linked Rha. (1→3)-β-Gal terminated with (1→3)-α-Ara and (1→3)-β-Rha. **LBP-a4:** 10,200 Da, Fuc:Gal = 0.41:1 **LBPC2:** 12,000 Da, Xyl:Rha:Man = 8.8:2.3:1. Heteropolysaccharide with (1→4), (1→6) β-glycosidic bond. **LBPC4:** 10,000 Da, Glc. Heteropolysaccharide with (1→4), (1→6) α-glycosidic bond.	[13,14,15]
*Herba cistanche*	*Cistanche deserticola* Ma.	Herba cistanche is composed of one neutral polysaccharide (CTZ) and five acidic polysaccharides **(CT1, CT2, CT3, CT4, and CT5)**, with contents of 299.2, 168.0, 123.2, 121.6, 54.4 and 11.2 mg/g, respectively.	[16]
*Sea-buckthorn*	*Hippophae rhamnoides* Linn.	Sea-buckthorn polysaccharides contain the neutral polysaccharide **SBP-I** and the acidic polysaccharides **SBP-II, SBP-III, and SP0.1-1.** **SBP-I** is composed of Ara, Xyl, Man, Glc, and Gal with a molar ratio of 1.18∶1∶2.20∶32.17∶1.45. **SBP-II** is composed of Xyl, Man, Glc, and Gal with a molar ratio of 1∶0.28∶1.02∶0.20. **SBP-III** consists of Xyl, Glc, and Gal with a molar ratio of 1∶2.15∶0.28. **SP0.1-1** is composed of Man, Glc, Gal, and Ara in a molar ratio of 1:2.3:1.9:11.2, with a core structure containing 1,4-linked-α-D-Glc; 1,4, 6-linked-α-D-Glc; and 1,4-linked-α-D-Man residues as the backbone. The side-chains are composed of 1,3,5-linked-α-L-Ara; 1,5-linked-α-L-Ara; terminal α-Ara; and 1,4-linked-β-D-Gal.	[17,18,19]
*Turnip*	*Brassica rapa* L.	Acidic polysaccharides in turnips consist of D-GalA, D-Man, L-Ara, D-Gal, D-Glc, L-Rha, D-GlcA = 67.73∶17.19∶10.20∶3.19∶1.11∶0.34:0.23. *Brassica rapa* L. acidic polysaccharide (BRAP): **BRAP-1:** Ara:Glc:Gal:GalA = 2.07:4.53:2.20:1; **BRAP-2:** Rha:Ara:Glc:Gal:GalA = 1.06:5.03:2.22:1.5.	[7,20]
*Chicory*	*Cichorium intybus* L.	The main components of chicory polysaccharides are a kind of fructan with similar structure. This fructan is a straight chain polysaccharide with a β-2, 1-glycosidic bond between fructose residues (F) and a glucose residue (G) at the end. The structural formula is G-1, (2-F-1) N-1, 2-F. In addition, chicory polysaccharides also contain a small amount of inulonose, that is, fructoses without a G terminal; the structure is F-1, (2-F-1) N-2, 2-F.	[21]
*Mulberry*	*Morus alba* L.	Three kinds of polysugars are separated from mulberry, namely, **MFP-1, MFP-2, MFP-3** and **MFP-4,** which are composed of Ara, Gal, Glc, Gly, Xyl, and GalA. **MFP3** glycosidic bond types include:(1→6)-linked α-D-Glc, (1→2)-linked α-L-Rha, (1→3)-linked α-D-Gal, (1→3)-linked β-L-Rha and (1→)-linked α-L-Ara.	[22,23]
*Garlic*	*Allium sativum* L.	Garlic polysaccharides are mainly composed of Ara, Gal, Glc, Xyl, Fru, and other monosaccharides.	[24,25,26]
*Basil*	*Ocimum basilicum* L.	Pure basil polysaccharide (OBP) is composed of Rha, Ara, Fuc, Alo, Man, and Gal, and the relative molar ratio is 2.18:4.025:2.38:0.15:1.7:0.357.	[27,28]
*Alfalfa*	*Medicago Sativa* Linn.	Alfalfa polysaccharide components include **APS-2a, APS-2b, APS-3a, APS-3b,** and **APPS.** **APS-3b** has the most complex monosaccharide composition. The monosaccharide composition and molar ratio are Xyl:Ara:Glc:Rha:Gal:GlcA:GalA = 1.00:2.35:3.78:3.05.2.74:1.72:11.45. **APS-3a** has the highest content of polysaccharide and uronic acid, at 92.88% and 52.09%, respectively. Pectic polysaccharide **(APPS)** is characterized to be a rhamnogalacturonan I (RG-I) type pectin with a molecular weight of 2.38 × 10^3^ Da and a radius of 123 nm. Primary structural analysis indicates that APPS is composed of a (1→2)-α-L-Rha-(1→4)-α-D-GalA-(1→2) backbone with 12% branching point at C-4 of Rha forming side chains by L-arabinosyl and D-galactosyl oligosaccharide units.	[29,30]
*Pomegranate*	*Punica granatum* L.	**Polysaccharide components of pomegranate peel I:** Ara, GalA, Gal, Rha, Glc, GlcA, Man. (Ara > GalA > Gal > Rha > Glc > GlcA > Man) **Polysaccharide components of pomegranate peel II:** Ara, Gal, Rha, Glc, GalA, Xyl, Man, and Fuc. (Ara > Gal > Rha > Glc > GalA > Xyl > Man > Fuc)	[31,32]
*Resina ferulae*	*Ferula sinkiangensis* K. M. Shen.	Total polysaccharides of Avegeron (FSPt): **FSP30, FSP50, FSP70, FSP80** Pleurotus ferulae lenzi polysaccharide: **PFLP1** contains four kinds of monosaccharide (Rha, Man, Glu, and Gal), in a molar ratio of 1:1.54:18.6:3.64; **PFLP2** contains five monosaccharides (Rha, Xyl, Man, Glu, and Gal), with a molar ratio of 1:1.08:0.65:6.76:4.28. **PFLP1:**[→3)-β-D-Glu-(1→3)-β-D-Glu-(1→3)-β-D-Man p-(1→3)-β-D-Glu-(1→3)-β-D-Glu-(1→3)-β-D-Glu-( 1→3)-β-D-Glu-(1→3)-β-D-Glu-(1→2)-β-D-Rha-(1 →3)-β-D-Glu-(1→3)-β-D-Glu-(1→3)-β-D-Glu-(1→ 3)-β-D-Glu-(1→3)-β-D-Glu-(1→3)-β-D-Man-(1→3) -β-D-Glu-(1→3)-β-D-Glu-(1→]_n_ **PFLP2:**[→3)-β-D-Glu-(1→3)-β-D-Glu-(1→4)-β-D-Xyl -(1→3)-β-D-Glu-(1→3)-β-D-Glu-(1→2)-β-D-Rha-(1 →3)-β-D-Glu-(1→3)-β-D-Glu-(1→3)-β-D-Man-(1 →3)-β-D-Glu-(1→]_n_	[33]
*Korla pear*	*Pyrus sinkiangensis* Yu.	Korla pear polysaccharides (PSP) are divided into Korla pear acidic polysaccharide (PSAP) and Korla pear neutral polysaccharide (PSNP). **PSNP-1:** Glc:Xyl:Gal = 3:1.6:1, in addition to a small amount of Ara, Fuc, and Man. **PSNP-2:** Glc:Xyl:Ara:Gal = 2.4:1:2.5:1.1, in addition to a small amount of Fuc and Man. PSAP-1 is mainly composed of GalA and contains a small amount of Ara and Rha. PSAP-2 is mainly composed of GalA and contains a small amount of Ara and Rha.	[34,35,36,37]
*Hops*	*Humulus Lupulus* L.	Hops polysaccharides: **HLP50-1, HLP50-2, HLP70-2-1, HLP70-2-2, HLP70-3, HLBP-2, HLBP-3,** and **HLBP-4** The molecular weights are 49.13 kDa, 73.25 kDa, 11.12 kDa, 7.3 kDa, 26.48 kDa, 6.13 kDa, 35.23 kDa, and 33.12 kDa, respectively.	[25]
*Fritillaria*	*Fritillaria walujewii* Regel	Fritillaria polysaccharide (FPSP) is composed of Xyl, Glc, Gal, and Man, with a molar ratio of 1: 58.02: 0.73	[38]
*Dandelion*	*Taraxacum altaicum* Schischk.	Dandelion polysaccharide: **TMP-1** without uronic acid is neutral and its purity is 95.2%. **TMP-2** contains uronic acid as an acidic polysaccharide, and its purity is 85.9%. Dandelion root polysaccharide (DRP): **DRP-2b:** with a molecular weight of 31.8 kDa, is composed of Rha, GlcA, Glc, Gal, and Ara; **DRP-3a:** with a molecular weight of 6.72 kDa, is composed of Rha, Glc, Gal, and Ara. The backbones of DRP-2b and DRP-3a are mainly composed of (1→5)-linked-α-D-Ara and (1→6)-linked-α-D-Glc, respectively.	[39,40]
*Lilium*	*Lilium martagon var. pilosiusculum* Freyn	Lilium polysaccharides are **LLPS-1, LLPS-2,** and **LLPS-3**. The average molecular weight of the three lilium polysaccharides are 350.5 kDa, 403.3 kDa, and 146.2 kDa, respectively.	[41,42]
*Selfheal*	*Prunella vulgaris* L.	Selfheal polysaccharides: **PV-P1, PV-P2, PV-P3.** **PV-P1** Mainly constitued by →5)-Ara-(1→,→3)-Xyl-(1 and 6)-Glc-(1- residue composition, and has a molar ratio of 125:29.1:3.2. In addition, it contains the terminal mannose, terminal galactose, 3-Gal-(1→,→4,6)-Glc (1→,and→3,6) -Gal-(1→ residue composition. **PV-P2** is mainly composed of terminal galacto, →3)-Xyl-(1→,→6)-Glc-(1→,→3)-Gal-(1→,→5)-Ara-(1→ with a molar ratio of 23: 8.1:29:3.5:4.1:38. **PV-P3** mainly consists of →3)-Xyl-(1→,→5)-Ara-(1→ and terminal mannose, **with** a molar ratio of 59:26:2.7.	[2]

**Table 2:** Similarities and differences in the medicinal and edible monosaccharides of five Xinjiang specialty plant polysaccharides.

Type	Monosaccharide	Chemical Formula	Molecular Weight	Structural Formula
Common	Ara	C_5_H_10_O_5_	150.13	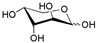
	Gal	C_6_H_12_O_6_	180.16	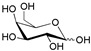
	Glc	C_6_H_12_O_6_	180.16	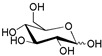
Different	Xyl (*Mulberry*, *Pomegranate*, *Kunlun snow chrysanthemum*)	C_5_H_10_O_5_	150.13	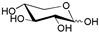
	Glc-A (*Turnip*, *Pomegranate*)	C_6_H_10_O_7_	194.14	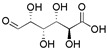
	Gal-A (*Mulberry*)	C_6_H_10_O_7_	194.14	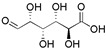
	Man (*Turnip*, *Pomegranate*)	C_6_H_12_O_6_	180.16	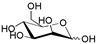
	Rha (*Pomegranate*)	C_6_H_12_O_5_	164.16	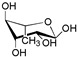
	Fuc (*Pomegranate*)	C_6_H_12_O_5_	164.16	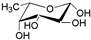

**Table 3:** Extraction and isolation methods of five plant polysaccharides.

Plants	Extraction Site	Extaction	Separation and Purification	Reference
*Turnip*	Root	Reflux extraction, Ethanol subsiding method	DEAE-cellulose, Sephadex G-100 column, Gel permeation chromatography (GPC)	[51]
			DAED-650M, Sepharese 6B, Sephacry1S-300	[20]
			DEAE-52 cellulose, Sephadex G-100 column	[7]
			HP-100 macroporous resin, DEAE-52 cellulose, Sephadex G-150 column	[52]
	Seed		-	[53]
*Kunlun snow chrysanthemum*	Flowers	Reflux extraction, Ethanol subsiding method	DEAE-650M, Sepharose-6B column (5.0 i.d. × 100 cm), Sephacryl S-300 HR column (2.2 i.d. × 90 cm), PL aquagel-OH MIXED-H column (7.5 m × 300 mm, 8µm) on an Agilent 1200 system,	[6]
			DEAE-52 Cellulose, Sephacryl S-100, Sephadex G-75 column	[20]
		Complex enzyme-ultrasonic-assisted extraction	DEAE-52 Cellulose ion exchange, Sephadex G-100 Dextran gel column	[54]
		Ultrasonic-assisted hot water extraction–alcohol precipitation method	DEAE-52 chromatography column, Sephadex G-100 gel chromatography column, UV spectroscopy, Freeze–thaw analysis, Sephadex G-100 gel column chromatography	[6]
*Pomegranate*	Seed	Enzymatic method (pectinase)	-	[31]
		Hot water extraction, ultrasonic-assisted water extraction and compound enzyme method	-	[32]
		Polyethylene glycol combined with ultrasonic microwave-assisted extraction technology	High-speed countercurrent chromatography(HSCCC), Gel permeation chromatography(GPC)	[50]
	Skin	Reflux extraction, Ethanol subsiding method	DEAE-cellulose column	[51]
*Mulberry*	Fruits	Reflux extraction, Ethanol subsiding method	DEAE-52 Cellulose column, Sephadex G-100 Dextran gel column	[24]
		Reflux extraction, ultrasonic-assisted extraction, Enzyme-assisted extraction, High-speed shear-technology-assisted extraction	-	[26]
		Oil ether reflux degreasing combined with water extraction and alcohol precipitation	AB-8 macroporous resin, DEAE cellulose column, SepharseCL-B gel column layer	[52]
*Jujube*	Fruits	Ethanol subsiding method	Anion exchange, Sepharose CL-6B column, High-performance gel permeation chromatography	[53]
		Ultrasonic extraction	Ion exchange, Gel permeation chromatography	[54]
			DEAE-52 cellulose column, Sephadex G-100 Dextran gel column	[55]
		Ethanol subsiding method	DEAE-52 cellulose column, Sephadex G-100 column	[54]
		Hot water extraction, Ultrasonic-assisted extraction	DEAE-52 anion exchange column, Sephadex G-100 column	[55]
		Cellulase method	-	[53]
	Leaf	Ultrasonic extraction	-	[8]

## Data Availability

Not applicable.
